# Dynamic resource allocation for controlling pathogen spread on a large metapopulation network

**DOI:** 10.1098/rsif.2021.0744

**Published:** 2022-03-09

**Authors:** Lina Cristancho-Fajardo, Pauline Ezanno, Elisabeta Vergu

**Affiliations:** ^1^ Université Paris-Saclay, INRAE, MaIAGE, Jouy-en-Josas 78350, France; ^2^ INRAE, Oniris, BIOEPAR, Site de la Chantrerie, CS40706, Nantes 44307, France

**Keywords:** infectious disease control, stochastic modelling, optimization, greedy scores

## Abstract

To control the spread of an infectious disease over a large network, the optimal allocation by a social planner of a limited resource is a fundamental and difficult problem. We address this problem for a livestock disease that propagates on an animal trade network according to an epidemiological–demographic model based on animal demographics and trade data. We assume that the resource is dynamically allocated following a certain score, up to the limit of resource availability. We adapt a greedy approach to the metapopulation framework, obtaining new scores that minimize approximations of two different objective functions, for two control measures: vaccination and treatment. Through intensive simulations, we compare the greedy scores with several heuristics. Although topology-based scores can limit the spread of the disease, information on herd health status seems crucial to eradicating the disease. In particular, greedy scores are among the most effective in reducing disease prevalence, even though they do not always perform the best. However, some scores may be preferred in real life because they are easier to calculate or because they use a smaller amount of resources. The developed approach could be adapted to other epidemiological models or to other control measures in the metapopulation setting.

## 1. Introduction

Infectious disease spread is a problem that can have important social, sanitary and economic consequences. Like for human diseases, this is a major public health concern for animal diseases, for guaranteeing animal welfare and food security [1]. In this context, epidemiological models, together with other relevant mathematical approaches, can help in the description and understanding of the mechanisms involved in disease propagation, as well as in assessing the effectiveness of control measures [2]. An approach for controlling a disease spreading on a population, from a social planner’s point of view, is the allocation of a resource that has an effect on this spread [3]. Many questions can arise in this context: how much resource is needed to restrain the disease propagation to a certain level [4–6], when should it be allocated [7] and where. In this work, we are interested in the third question. More specifically, we are concerned with the problem of dynamically deciding where to allocate a limited available resource in an optimal manner, in order to minimize disease spread on a large animal metapopulation network.

On the one hand, most of the research addressing the issue of resource optimal allocation on a large network [8–12] does not focus on metapopulation networks, i.e. does not account for infection-related dynamics within each sub-population represented by a node of the network. Even more, works relying on mean-field theory [13] do not consider structured populations, in particular as a network.

On the other hand, the existing studies addressing the resource allocation problem on metapopulation networks are based on techniques that lack of scalability. For example, optimal control [14] and reinforcement learning [15], which would theoretically give an optimal strategy, cannot be used in the context of very large networks due to the dimensionality in the state space [16,17]. Even if we consider a sub-population as healthy or infected (only two possible states per sub-population), a network with *J* sub-populations would have 2^*J*^ possible health states, which yields an asymptotically intractable optimal allocation when the network is very large. Hence, authors that study resource allocation on metapopulation networks generally build and evaluate their approach in a small number of sub-populations, usually less than 50 [18–25]. Recently, [26] proposed a framework built upon optimal control theory that is able to deal with the dynamic allocation problem in a network of hundreds of sub-populations thanks to several simplifications, among which is considering only a subset of edges for the optimization. Yet, such a scale does not allow one to capture the structural characteristics of complex networks, such as the animal trade network we consider here. In particular, animal trade networks are in general scale-free [27] (most herds have few trading partners while a few herds have many) and dynamic, as the amount of exchanged animals can vary over time [28].

Finally, published studies assume the resource to be in general distributed only once, before or at the beginning of an outbreak [21,29]. Therefore, the resource allocation problem is static. However, the allocation problem can be intrinsically dynamic if it is studied in the long run. For example, if the available resource is a vaccine, this can have a limited effect in time, so there is need for several vaccination campaigns.

Given the intractability of the optimal strategy, in this work we restrict ourselves to score-based strategies, i.e. strategies that consist of allocating the resource according to a scoring function (or indicator), up to the limit given by the available quantity of the resource. Furthermore, given the practical importance of dynamical aspects of the allocation problem, we include this view in the present study, i.e. we assume that the resource allocation is dynamic.

The contribution of this work is twofold. First, from a methodological perspective, by adapting the greedy approach in [30], we obtain analytic scores for controlling disease spread on a large animal trade network, where the disease propagation is represented by a stochastic SIR model that accounts for demography and trade (model introduced in §2.1). The approach consists of finding the scoring function that minimizes a short-term approximation of a given objective function (§§2.2, 2.3.1 and 3.1). Our generalization is mainly driven by the metapopulation framework, which implies that a herd is not only infected or healthy, but that it has an internal infection and demographic dynamics. In particular, this allows for the possibility of needing different amounts of resources for different herds. Furthermore, we extend this approach for two different types of resources (vaccination and treatment) and two different objective functions (the number of infected animals and the number of infected herds). Second, regarding real-life disease control for livestock diseases, in §3.2, we evaluate the performances of the analytically obtained strategies along with the one of several heuristic strategies (introduced in §2.3.2) that can be relevant for this context. Finally, in §4, we extend the interpretation of the analytically found scores and discuss their suitability in a metapopulation context in the light of simulation-based results. We also consider several perspectives, based either on the development of other greedy scores or on new simulation studies that could use the explorations performed in this work.

## 2. Methods

### 2.1. SIR stochastic epidemiological model with demography in a metapopulation based on a trade network

**Figure 1.**
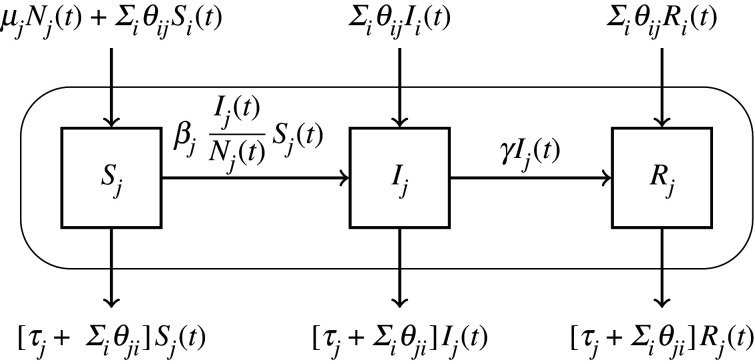
Schematic representation of the intra-herd infection and demographic dynamics for a herd *j*, without resource allocation. Horizontal arrows represent transitions between health-related compartments, corresponding to the course of infection inside the herd (curved rectangle), while vertical arrows represent animal flows to and from the herd. Coefficients on the arrows are transition rates. See main text in §2.1 for parameter definitions.

We considered a livestock disease that spreads on a large animal trade network composed of *J* herds. We supposed that the disease introduction in a herd could only be due to animal transfers, and that it could only be transmitted between animals of the same herd. This livestock trade network underlies a metapopulation network where nodes represent herds and links represent animal transfers from one herd to another. For representing this system, we used the stochastic intra-herd SIR epidemiological–demographic model described in [31], which takes into account animal exchanges. The model is summarized in figure 1. *S*_*j*_(*t*), *I*_*j*_(*t*) and *R*_*j*_(*t*) are the number of susceptible, infected and recovered animals in herd *j* at time *t*. Parameters *β*_*j*_, *μ*_*j*_ and *τ*_*j*_ are the daily rates of disease transmission, birth and death in herd *j*, assuming newborns are all susceptible. As for *γ*, it is the daily recovery rate from the infection, reasonably assumed equal for all herds. Finally, *θ*_*ji*_ is the daily out rate of animals going from herd *j* to herd *i*, assuming animals in any health state can be exchanged. We denote as *N*_*j*_(*t*) the size of herd *j* at time *t*. The model was specified as a continuous-time Markov chain, and its simulation was built on an Euler discrete-time scheme using multinomial distributions, as described in [32]. Details can be found in the electronic supplementary material of [31].

### 2.2. Dynamic resource allocation problem in the metapopulation framework

We supposed that there is a central social planner seeking to minimize the disease propagation on the animal trade network, by distributing a limited amount of a resource among the herds in the network, dynamically with a given decision time-step. This dynamic resource allocation problem was formulated as:

2.1 $$\min_{A}F(A)\;\text{subject to }\sum_{\;j=1}^{J}b_{j}(t)A_{j}(t)\leq b_{\mathrm{fix}},\;\forall t=\Delta_{d},2\Delta_{d},\ldots$$

*F*(*A*) in equation (2.1) is the function that the planner has to minimize, which depends on the allocation strategy *A*, i.e. the function that determines the decisions $A_{j}(t),\forall j=1,\ldots ,J,$ $\forall t=\Delta_{d},2\Delta_{d},\ldots$, where *j* denotes the herd, and Δ_*d*_ is the decision time-step. We assumed binary allocation decisions for each herd, i.e. *A*_*j*_(*t*) = 1 if the resource is allocated to herd *j* at time *t*, *A*_*j*_(*t*) = 0 otherwise. In the condition of equation (2.1), *b*_fix_ is the quantity of resource that is available at each decision time and *b*_*j*_(*t*) is the quantity of the resource that would be needed for herd *j* at time *t* if this herd was selected for the allocation.

We supposed that the resource to allocate could be either a vaccine or a treatment. For the vaccine, we assumed that if applied to a susceptible animal at time *t*, the disease transmission rate towards this susceptible animal becomes *β*^*v*^ = *β*(1 − *e*_*v*_) during the period ]*t*; *t* + Δ_*d*_], where 0 ≤ *e*_*v*_ ≤ 1 is the protective efficacy of the vaccine. That is, Δ_*d*_ is also the duration of the vaccine’s efficacy. We underline that even if all susceptible animals in herd *j* are vaccinated at time *t*, infections can occur within *j* from time *t* to *t* + Δ_*d*_ if new susceptible animals enter the herd, through births or imports of susceptible animals from other herds. For the treatment, we supposed that it increases the recovery rate of treated infected animals by an additional factor *γ*′, i.e. reducing the mean duration of the infectious period for these animals to (*γ* + *γ*′)^−1^.

Regarding the resource constraint, for vaccination we assumed it concerned the number of available doses, and if a herd *j* was selected at time *t* all the animals in the herd would be vaccinated. So in the condition in equation (2.1), *b*_*j*_(*t*) = *N*_*j*_(*t*) (the size of herd *j* at time *t*). For the treatment, the resource constraint was on the number of herds in which animals were treated at each decision time, so *b*_*j*_(*t*) = 1. The choice to consider that the constraint for the treatment involves the number of herds was mainly motivated by analytical considerations discussed in §2.3.1.

### 2.3. Score-based strategies

**Figure 2.**
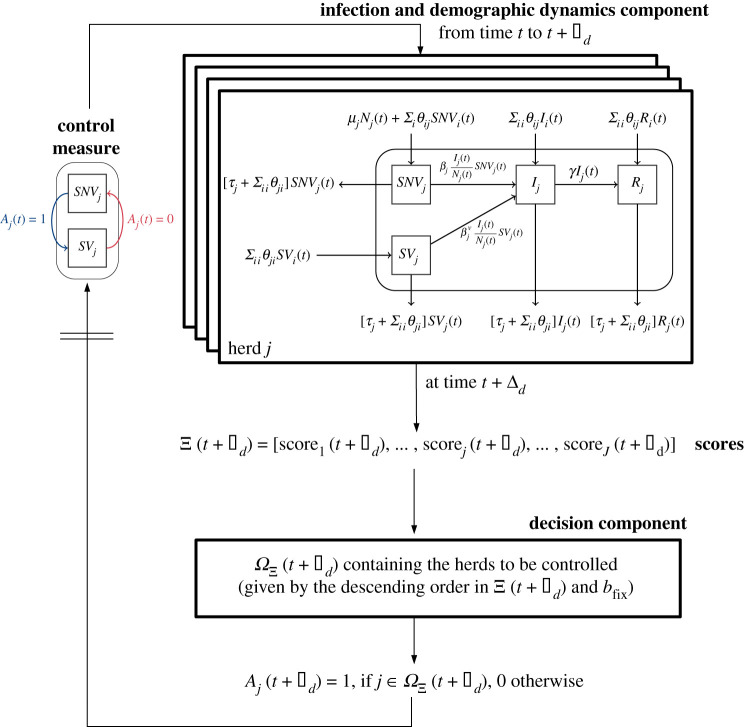
Representation of the modelling and optimization framework of the score-based dynamic resource allocation under constraint, applied to a metapopulation and vaccine allocation. See main text in §§ 2.1 and 2.2 for parameter definitions.

Score-based strategies consist of ordering herds according to a certain scoring function Ξ and selecting the top herds, up to the limit given by the condition in equation (2.1). Let $\Omega_{\Xi }(t)$ be the set that contains the selected herds according to Ξ(t) (the score values at time *t*) and *b*_fix_ (the available quantity of resource per decision time-step). Then, *A*_*j*_(*t*) = 1 if $j\in \Omega_{\Xi }(t)$, 0 otherwise. Figure 2 represents the modelling and optimization framework of the dynamic resource allocation of vaccines under a score-based strategy. The treatment allocation differs only in the infection and demographic dynamics component, and in the control measure component. That is, for any herd *j*, there is an additional compartment *T*_*j*_ for treated infected animals, where animals go from *I*_*j*_ to *T*_*j*_ at decision time *t*, if *A*_*j*_(*t*) = 1. Unlike vaccination, the transition from *T*_*j*_ to *I*_*j*_ is not possible even if *A*_*j*_(*t*) = 0, i.e. treated animals can only recover.

Regarding the scoring function, it can be either optimized or heuristic. In the following, we address the two possibilities.

#### 2.3.1. Greedy scores

First, following the approach in [30] we searched for optimized scoring functions. This approach consists of finding a scoring function that minimizes a short-term approximation of the objective function

$$F(A)\;:=\int_{0}^{\infty }\;e^{-at}E[J_{I}^{A}(t)]\;dt,$$

where $J_{I}^{A}(t)$ is a function of the infection-related state of the network at time *t*. The objective establishes an infinite time horizon, and a parameter *a* ≥ 0 which reduces the long-term impact of $J_{I}^{A}(t)$. This means that the larger *a*, the more we are interested in the short-term behaviour of the infection dynamics, i.e. the more weight we put on the initial stochasticity of the disease diffusion.

Let us deote by *X*(*t*) the infection-related state of the network at time *t*. In the SIR metapopulation framework, *X*(*t*) is a *J* × 3 matrix where each row *j* ∈ {1, …, *J*} contains the values of *S*_*j*_(*t*), *I*_*j*_(*t*) and *R*_*j*_(*t*) at time *t*. Since the allocation strategy has no impact on the initial state, and since the process is Markovian, the minimization problem (equation (2.1)) is equivalent to

2.2 $$\min_{A}\int_{u=0}^{\infty }\;e^{-au}\;E[J_{I}^{A}(t+u)|X(t)=X]\;du,$$

for all decision times *t* and for all network possible states *X*, under the same constraint in equation (2.1). Then, focusing on the short-term behaviour of the system, it is possible to obtain an approximation of the objective function, which is based on a Taylor expansion of $E[J_{I}^{A}(t+u)|X(t)=X]$ for a small value of *u*. Finally, one must find the scoring function that minimizes this approximation. This approach is therefore called greedy, as it yields locally (i.e. short term) optimal allocation decisions.

We stress that the form of the scoring function will not depend on the parameter *a* in equation (2.2), i.e. we do not need to fix a value for this parameter when exhibiting the scoring function. Yet, the higher is *a*, the lower is the impact of the approximation accuracy. See the electronic supplementary material, S1, for more details on the approach.

We adapted this greedy approach to the metapopulation framework by considering, for each one of the two types of resource (vaccination and treatment), the minimization of two different objective functions in equation (2.2). A function on the number of infected animals

2.3 $$J_{I}^{A}(t)=\sum_{\;j=1}^{J}I_{j}(t),$$

and a function on the number of infected herds

2.4 $$J_{I}^{A}(t)=\sum_{\;j=1}^{J}1_{I_{j}(t)>0}.\;$$

That is, we treated four different cases, depending on the type of resource (vaccine or treatment) and the objective to be minimized: the number of infected animals (equation (2.3)) or the number of infected herds (equation (2.4)). In particular, the derivation of the score for the allocation of a treatment under the objective of equation (2.4) required to consider that the resource constraint of equation (2.1) was expressed in terms of the number of attainable herds (*b*_*j*_(*t*) = 1). This allowed the total number of treated herds to be formulated as the minimum between *b*_fix_ and the number of herds that have exactly one infected animal (see electronic supplementary material, S1.2.1, for details).

#### 2.3.2. Heuristic scores

In addition, we considered three types of heuristic scores based on: the topology of the static aggregated network; the demographic changes in the network; and the dynamic infection-related state of the network. Table 1 contains the list of the 16 heuristic scoring functions we tested for the metapopulation framework: five topological ones (in-strength, out-strength, closeness, betweenness and pagerank), five demographic ones (*N*_*j*_(*t*), purchases_*j*_(0, *t*), purchases_*j*_(*t* − Δ_*d*_, *t*), sales_*j*_(0, *t*), sales_*j*_(*t* − Δ_*d*_, *t*)), 5 epidemiological ones (*s*_*j*_(*t*), *i*_*j*_(*t*), *r*_*j*_(*t*), *i*_*j*_(*t* − Δ_*d*_, *t*), *r*_*j*_(*t* − Δ_*d*_, *t*)), and a random scoring function. All the topological scoring functions are classical centrality measures in networks [33].

**Table 1.** Heuristic scoring functions for herd *j* at time *t*. Dependence on *t* means the score is dynamic in time, otherwise it is static.

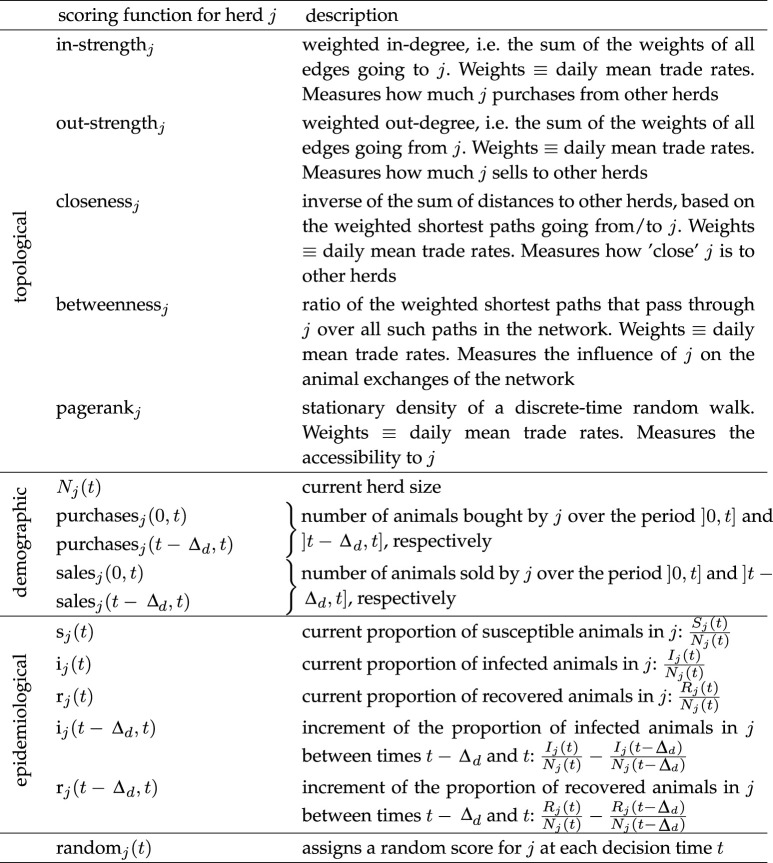

### 2.4. Simulation setting and numerical explorations

The simulation setting was described in detail in the electronic supplementary material of [31]. The metapopulation structure was set close to real data, extracted from the French Cattle Identification Database (FCID): animal movements correspond to the Finistère administrative area in western France, which is densely populated with cattle. The trade parameters ($\theta_{\;ji},\;\forall j,i=1,\ldots ,J$) underlying the network structure were set to values based on these data. The network is scale-free and consists of *J* = 5000 herds. The initial herd size distribution can be found in the electronic supplementary material, figure S1*a.* Details on the trade parameters and on the herd size distribution can be found in electronic supplementary material, fig. S3 of [31].

Without loss of generality, the values of demographic and epidemiological parameters were set to the same value for all herds. In particular, death and birth rates were $\tau_{j}=\tau ,\mu_{j}=\mu ,\;\forall j=1,\ldots ,J$, where *τ* = 0.0009 days^−1^ and *μ* = 2*τ*. So, based on field reality, animals have a mean life time of approximately three years, and on average an animal gives birth to a calf every 1.5 years. As herd sizes could vary not only through births and deaths but also through animal movements, we established a constraint on the capacity of each herd, *N*_*j*_(*t*) ≤ 1.5 *N*_*j*_(0), so that these remain relatively stable over time. Electronic supplementary material, figure S1*b*, shows the final herd size distribution for a run of the epidemiological–demographic model (without resource allocation) on the simulated trade network. Regarding the epidemiological parameters, we set $\beta_{j}=\beta ,\;\forall j=1,\ldots ,J$, and considered a disease with moderate immediate impact and long-lasting development (*β*/*γ* = 2 and 1/*γ* = 90 days). A second numerical setting, corresponding to a disease with higher early peak and smaller infection duration (*β*/*γ* = 4, 1/*γ* = 30 days), was explored in the electronic supplementary material, S2. Finally, for the available resource, we assumed it could either be a perfectly effective protective vaccine, i.e. *e*_*v*_ = 1, or a treatment that greatly reduces the infectious period of infected animals (but which is not perfect in order to avoid instantaneous recovery, an unrealistic assumption). More specifically, the duration of the infectious period with treatment was assumed to be 3% of the duration without treatment, i.e. the mean duration of the infectious period for a treated infected animal is (*γ* + *γ*′)^−1^ = 0.03(1/*γ*) = 2.7 days.

#### 2.4.1. Setting for the exploration of infection-related dynamics with score-based resource allocation

Given these parameter values, we simulated the infection-related dynamics of the metapopulation during 3 years in 74 ( = ([16 + 3] + [16 + 2]) × 2) cases characterized by the type of resource, the score according to which it is allocated (16 × 3 for vaccination and 16 × 2 for treatment) and the scenario. This last one can be: an *epidemic* scenario, where initially 10% of the herds (chosen completely at random) had a random subset of 15% of their animals infected; or an *endemic* scenario, where the initial state was given by the state at roughly 3 years (1080 days) without resource allocation departing from the epidemic scenario. Indeed, electronic supplementary material, figure S9, shows that if the simulations are extended beyond 3 years, the total proportion of infected animals remains rather stable, and that there is only a 10% reduction in the proportion of infected herds between levels attained at 3 and 9 years. Hence, although the infection dynamics after 3 years of simulation did not reach a steady state rigorously speaking, this date was chosen as the initial point of the endemic scenario. Indeed, on the one hand, at this date the pathogen had widely spread in the metapopulation (electronic supplementary material, figure S9), and on the other hand, considering 3 years limited the simulation cost. In each case, we explored the dynamics of the proportion of infected herds and of the total number of infected animals for a fixed value of the available quantity of resource, *b*_fix_. The values of *b*_fix_ and Δ_*d*_ parameters can be found in table 2. We supposed that vaccination decisions were more spaced in time since vaccines are preventive and tend to have long-lasting effect. Meanwhile, we supposed that treatment decisions were more frequent as they are more prone to being applied in a critical situation.

**Table 2.** Parameter values in the allocation problem depending on the type of resource.

resource	parameter	definition	values
vaccine	*b*_fix_	number of available doses at each decision time (as a % of the initial total number of animals)	($25\text{\%}\times \sum_{\;j=1}^{J}N_{j}(0)$)^a^ and $([5\text{\%}-90\text{\%}]\times \sum_{\;j=1}^{J}N_{j}{\left(0)\right)}^{b}$
	Δ_*d*_	decision step (in days)	180
treatment	*b*_fix_	number of attainable herds at each decision time	25^a^ and [5 − 100]^c^
	Δ_*d*_	decision step (in days)	15

^a^Values for infection-related dynamics explorations.

^b^Values for percolation analysis of vaccination. From 5% to 30%: by 0.5%. From 40% to 90%: by 10%.

^c^Values for percolation analysis of treatment. From 5 to 75: by 5. From 80 to 100: by 10.

We stress that in all simulations for the vaccine allocation, only herds with at least one susceptible animal were eligible, and that the treatment allocation only considered herds with at least one infected animal.

Furthermore, we evaluated how other quantities of interest, raw proxies of ‘costs’, varied with the chosen scoring function and the amount of available resource. We stress that the quantity of resource that is actually used may be less than the available quantity (*b*_fix_). For vaccination, we explored the mean proportion of wasted doses over time, i.e. the total number of vaccine doses allocated to infected or recovered animals, out of the number of available doses. For the treatment, where we assumed that only infected animals were treated, we evaluated the mean number of used doses over time, and we explored the mean size of the target population, i.e. the total number of animals in treated herds.

#### 2.4.2. Setting for percolation analysis

We performed percolation experiments by assessing the final (after 3 years) proportion of infected herds when the allocation was done using a given scoring function, and a fixed quantity of resource. We also included in this analysis an exploration of the raw proxies of costs. The range of values tested for *b*_fix_ (cf. table 2) was chosen so as to exacerbate differences in the infection-related dynamics by scoring function. The objective was to analyse the dependence of the relative performances of the scoring functions on limiting disease spread with respect to the available quantity of the resource. To avoid increasing computation time, we chose to run the percolation analysis only for a subset of scoring functions. This subset was determined from the results of the complete exploration setting described in §2.4.1.

Finally, we investigated the sensibility of the infection-related results of percolation analysis to the value of the vaccine efficacy *e*_*v*_. More specifically, we evaluated through simulations two cases where vaccine efficacy is not perfect: *e*_*v*_ = 0.9 and *e*_*v*_ = 0.7. Lower values for *e*_*v*_ were not considered, since it is very unlikely that a vaccine for a livestock disease with an efficacy below 0.7 is even considered in the field, as its implementation could induce greater economic costs than epidemiological benefits. With each of these values we ran the percolation analysis described above, for three values of the available number of doses: [25%,40%,70%] of the initial total number of animals in the metapopulation.

## 3. Results

### 3.1. Greedy scoring functions

We obtained four different analytic scoring functions by considering the minimization problem in equation (2.2) for each resource and for each objective with the greedy approach. The scoring functions for an objective on the number of infected herds (greedyV_infherds and greedyT_infherds) were obtained through a second-order Taylor expansion, while the other two scoring functions (greedyV_infanimals and greedyT_infanimals) were obtained using a first-order approximation. Details on how the greedy scoring functions were computed in each case can be found in the electronic supplementary material, S1.

As mentioned in the previous section, the values of *β*_*j*_, *τ*_*j*_, *μ*_*j*_ were set equal for all herds in our simulations, so we present in table 3 the scores in this setting. The greedy scoring functions found for the generic framework, where these values can be different across herds, can be found in electronic supplementary material, table S1. In simulations, five scoring functions (directly or indirectly issued from the greedy approach) were used. Table 3 includes in total six scoring functions: the four obtained by optimization, an additional scoring function for vaccination (greedyV_infherds_threshold) and the scoring function greedyT_infherds_threshold for the treatment, which replaced greedyT_infherds in our simulations.

**Table 3.** Greedy scoring functions studied in the numerical explorations. All the greedy scores are dynamic.

resource	$J_{I}^{A}(t)$	scoring function for herd *j*	scoring function name
vaccine	inf. animals (equation (2.3))	(*I*_*j*_(*t*)/*N*_*j*_(*t*))*S*_*j*_(*t*)	*greedyV_infanimals*
	inf. herds (equation (2.4))	$\left(I_{j}(t)/N_{j}(t))S_{j}(t)(1_{I_{j}(t)=1}(\gamma +\tau +\sum_{i\neq j}^{J}\theta_{\;ji})+\sum_{i\neq j}^{J}\theta_{\;ji}1_{I_{i}(t)=0}\right)$	*greedyV_infherds*
	inf. herds (equation (2.4))	$\left(I_{j}(t)/N_{j}(t))S_{j}(t)(1_{0<I_{j}(t)<20}(\gamma +\tau +\sum_{i\neq j}^{J}\theta_{\;ji})+\sum_{i\neq j}^{J}\theta_{\;ji}1_{I_{i}(t)=0}\right)$	*greedyV_infherds_threshold*
treatment	inf. animals (equation (2.3))	*I*_*j*_(*t*)	*greedyT_infanimals*
	inf. herds (equation (2.4))	${\left[-\sum_{i\neq j}^{J}\theta_{\;ji}1_{I_{i}(t)>0}\right]}_{\;j\;:\;I_{j}(t)=1}$	*greedyT_infherds*
	inf. herds (equation (2.4))	$\left[(-\sum_{i\neq j}^{J}\theta_{\;ji}1_{I_{i}(t)>0})1_{0<I_{j}(t)<20}\right]$	*greedyT_infherds_threshold*

To minimize the number of infected animals by distributing a vaccine, the greedyV_infanimals scoring function privileges herds with a large within-herd incidence rate (*βI*_*j*_(*t*)*S*_*j*_(*t*)/*N*_*j*_(*t*)), i.e. many infected animals and a large proportion of susceptible animals. For the treatment, with greedyT_infanimals the allocation would be made only as a function of the number of infected animals by herd (*I*_*j*_(*t*)).

Regarding the minimization of the number of infected herds, for vaccination it led to a scoring function, greedyV_infherds, favouring two types of herds: either herds that have a large within-herd incidence rate, and that send to many healthy herds (large $\sum_{i\neq j}\theta_{\;ji}1_{I_{i}(t)=0}$); or herds with only one infected animal, also presenting a large proportion of susceptible animals and which sell many animals (large $\sum_{i\neq j}^{J}\theta_{\;ji}$ ). With the same objective for treatment, the greedyT_infherds scoring function only concerns herds with exactly one infected animal, and among these, the priority is on herds that send the smallest flows to infected buyers (small $\sum_{i\neq j}^{J}\theta_{\;ji}1_{I_{i}(t)>0}$).

We remark that the minimization of the function on the number of infected animals for vaccination led to scoring functions that only depend on the epidemiological state of herd *j*, but not on the states of other herds, and in particular not on the topology of the network. This is due to the use of a first-order Taylor development for approximating the objective function (see electronic supplementary material, S1, for details).

The two additional scoring functions, considered on the basis of the analytically obtained scores, were built in the following way. For vaccination, the additional scoring function greedyV_infherds_threshold consists of replacing $1_{I_{j}(t)=1}$ by $1_{0<I_{j}(t)<20}$ in greedyV_infherds. This intends to avoid that the first term of the sum in the scoring function becomes 0 for herds that have few infected animals but not necessarily just one. Similarly, for the treatment, we replaced greedyT_infherds by a scoring function with a softer condition on the number of infected animals. The condition in greedyT_infherds on having exactly one infected animal for a herd to be eligible appeared to be too restrictive. Indeed, if the quantity of available treatment exceeded the number of herds that satisfy this condition, the rest of the treatment would not be allocated to any herd. Yet, allocating the exceeding treatment to herds with more than one infected animal could only be beneficial for limiting disease spread and would satisfy the constraint on the quantity of available treatment. Hence, the greedyT_infherds_threshold scoring function considers all herds that are potentially eligible with a non-negligible probability, yet favours herds that have few infected animals ($1_{0<I_{j}(t)<20}$).

### 3.2. Results of numerical explorations

#### 3.2.1. Infection-related dynamics following score-based resource allocation

**Figure 3.**
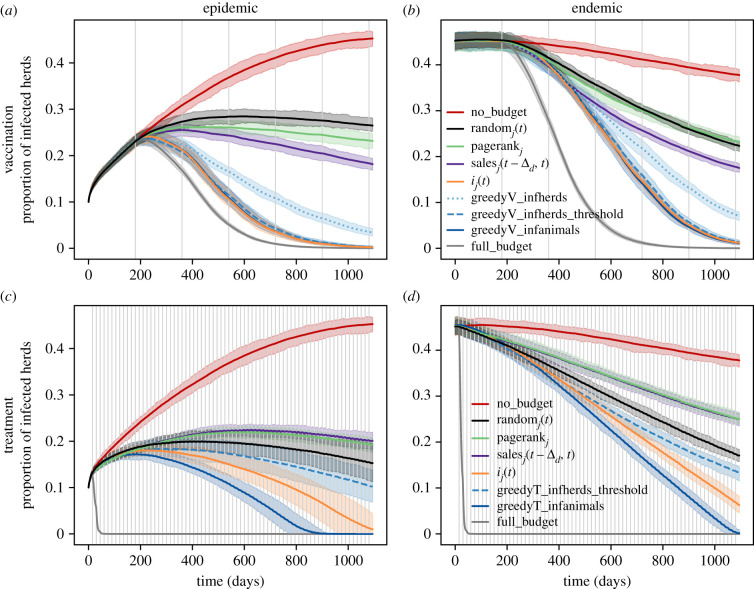
Dynamics of infection spread (proportion of infected herds) under resource allocation based on different scoring functions. Epidemic (left) and endemic (right) scenarios, for vaccination (*a*,*b*) and treatment (*c*,*d*). no_budget (red) represents the case where there was no resource allocated, and full_budget (grey) the case where the resource was not limited. For other curves, each colour represents the scoring function according to which the allocation decisions were taken: random (black), topological (green), demographic (purple), epidemiological (orange) and greedy scoring functions (blue tones). For vaccination, the amount of available doses by decision time was $b_{\mathrm{fix}}=25\text{\%}$ of the initial total number of animals in the metapopulation. For treatment, the number of attainable herds by decision time was *b*_fix_ = 25 herds. Grey vertical lines represent resource allocation times. Mean results and 90% confidence bands over 50 runs.

Figure 3 presents the results for the dynamics of the proportion of infected herds, under the setting described in §2.4.1 for a subset of the scoring functions. In particular, since the results for the topological scores were very similar, we present only the results of the pagerank_*j*_ score. This was also the case for demographic scores, so we chose the sales over the decision period, sales_*j*_(*t* − Δ_*d*_, *t*), as the representative score for this group. For the epidemiological scores, we present only the results for the best performing score, *i*_*j*_(*t*). In addition, we included as the best and worst reference cases, results for cases where there was sufficient resource for all herds (*full_budget*), and where there was no resource to allocate (*no_budget*). Electronic supplementary material, figure S2, presents the complete results for cases by scenario (epidemic or endemic), type of available resource (vaccine or treatment) and the heuristic or greedy score according to which the allocation was performed. We also included in electronic supplementary material, figure S2, results for the dynamics of the total number of infected animals, yet we remark they were similar to the ones found for the proportion of infected herds.

One of the main remarks emerging from the analysis of figure 3 and electronic supplementary material, figure S2, is that topological and demographic scoring functions were the groups that performed less well for limiting the disease spread, both for vaccination and for the treatment. This is in comparison with the group formed by the scoring functions that take into account the health statuses of the herds, i.e. the greedy scoring functions and the best performing epidemiological functions, in particular *i*_*j*_(*t*).

Furthermore, electronic supplementary material, figure S2, shows that for vaccination, the scoring function *s*_*j*_(*t*) (the proportion of susceptible animals) performed the worst for allocating the available quantity of vaccines (equal to 25% of the initial number of animals in the metapopulation) in both scenarios. The group of topological scoring functions performed better than the random scoring function in the epidemic scenario, but was not very different from it in the endemic scenario. Demographic scoring functions performed in general better than the topological ones, in particular sales_*j*_(0, *t*) and sales_*j*_(*t* − Δ_*d*_, *t*). Apart from *s*_*j*_(*t*), the epidemiological scoring functions had a good performance, except for the proportion of recovered animals (*r*_*j*_(*t*)) in the endemic scenario, where it was just as good as the random score.

The second important remark (figure 3) is that the best performing scores were the proportion of infected animals (*i*_*j*_(*t*)), and greedyV_infanimals. Electronic supplementary material, figure S2 shows that this applies both for reducing the total number of infected animals and the proportion of infected herds. They were closely followed by greedyV_infherds_threshold in both scenarios. Meanwhile, greedyV_infherds was not as good as these three, particularly in the endemic scenario.

Figure 3 and electronic supplementary material, figure S2, also present results when the resource was a treatment and *b*_fix_ was equal to 25 herds. In particular, electronic supplementary material, figure S2, shows that in the epidemic scenario, *s*_*j*_(*t*) performed badly when compared to the other scoring functions. Yet *r*_*j*_(*t*) arrived to perform worse at the end of the 3 years in this scenario, and was the worst-performing scoring function in the endemic scenario. Even more, these two scoring functions, the topological and demographic ones, and the difference in the proportion of recovered animals (*r*_*j*_(*t* − Δ_*d*_, *t*)), all performed worse than the random score in both scenarios. Regarding the other scoring functions, greedyT_infanimals (allocating according to the number of infected animals by herd, *I*_*j*_(*t*)) had the best performance in both scenarios. It was followed by the score *i*_*j*_(*t*), i.e. the proportion of infected animals by herd, though this last one did not manage to eradicate the disease before the 3 years. The next best performance was given by *i*_*j*_(*t* − Δ_*d*_, *t*), which was followed by greedyT_infherds_threshold. To sum up, for the treatment allocation, only the greedy scores and two epidemiological scores (*i*_*j*_(*t*) and *i*_*j*_(*t* − Δ_*d*_, *t*)) performed better than the random score. In particular, greedyT_infanimals was the only one that eradicated the disease within the 3 years (figure 3).

The dynamics of raw proxies of costs can be found in electronic supplementary material, figure S3. In particular, it is shown that the proportion of herds that were vaccinated varied according to the allocation scoring function. Indeed, topological and demographic scoring functions led to vaccinating slightly fewer herds than the epidemiological and the greedy scores (excluding *s*_*j*_(*t*)). Unsurprisingly, *s*_*j*_(*t*) led to the highest proportion of vaccinated herds and did not waste any doses, i.e. only vaccinated herds without infected or recovered animals, while *r*_*j*_(*t*) wasted the highest proportion of available doses. For the treatment, the topological and demographic scoring functions led to the smallest number of used doses and to the highest size of target population, contrary to the epidemiological and greedy scores.

Finally, electronic supplementary material, figure S4, shows the relationship between allocation decisions among different scoring functions at a given decision time. Both for vaccination and treatment, decisions according to the topological and demographic indicators were very similar (in terms of targeted herds) according to the Jaccard index [34]. For vaccination, decisions according to epidemiological and greedy scoring functions were similar at the first decision time (six months), yet this similarity diminished over time. Regarding decisions through time for a given scoring function (electronic supplementary material, figure S5), topological functions tended to allocate the resource to the same herds over time. This was also the case for demographic functions, except for the sales scoring functions in vaccination, for which the first decisions were less and less similar to decisions at the following decision times. On the contrary, for *s*_*j*_(*t*) the similarity between consecutive vaccination decisions seemed to increase over time. And for each of the other epidemiological and greedy scoring functions, vaccination decisions were in general less similar over time. Treatment decisions according to each epidemiological and greedy scoring function were very different over time as long as the disease was not eradicated, except for decisions according to *r*_*j*_(*t*).

#### 3.2.2. Percolation analysis results

**Figure 4.**
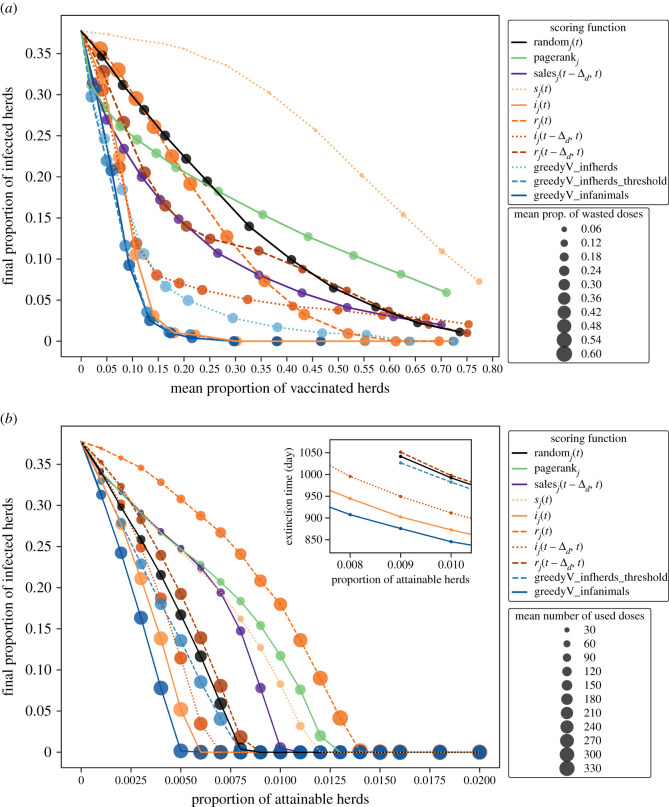
Percolation experiments results in the endemic scenario. Each colour represents the scoring function according to which the allocation decisions were taken: random (black), topological (green), demographic (purple), epidemiological (orange tones) and greedy scoring functions (blue tones). For (*a*) vaccination, final (after 3 years) mean (over runs) proportion of infected herds as a function of the mean (over time and over runs) proportion of vaccinated herds. Each point corresponds to a value of *b*_fix_, and its size represents the mean (over time and over runs) proportion of wasted doses. For (*b*) treatment, final (after 3 years) mean (over runs) proportion of infected herds as a function of the proportion of attainable herds at each decision time. Each point corresponds to a value of *b*_fix_, and its size represents the mean (over time and over runs) number of used doses. The inset shows the mean (over runs) extinction time with each scoring function, for a restricted range of the proportion of attainable herds. Results for 50 runs.

Figure 4 shows results of the percolation analysis in the endemic scenario for each type of resource, using a selected subset of scoring functions. Results in the epidemic scenario can be found in electronic supplementary material, figure S6. We ran this analysis using all the epidemiological and greedy scores, since the infection-related dynamics results in §3.2.1 were quite different for the scores within each of these groups. By contrast, because the results of the topological scores were very similar, as were the results of the demographic scores, we considered only one of each type: sales_*j*_(*t* − Δ_*d*_, *t*) for the demographic scoring functions and pagerank_*j*_ for the topological scores. This figure (also figure 4) confirms the main observations made in §3.2.1: certainly, the best-performing scoring functions for reducing disease prevalence, for almost every quantity of resource that we tested, were the greedy scores along with some epidemiological scores.

For vaccination, figure 4*a* shows that when the proportion of vaccinated herds was at least 0.10 of the initial number of animals in the metapopulation, the scoring functions that led to the lowest final proportion of infected herds were greedyV_inf_animals, greedyV_infherds_threshold and *i*_*j*_(*t*). They were followed by the greedyV_infherds scoring function. The topological and demographic scoring functions, pagerank_*j*_ and sales_*j*_(*t* − Δ_*d*_, *t*), were just as good as the greedy scores only when the available quantity of resource was very low (particularly when the proportion of vaccinated herds was less than 0.05), but did not perform well as this proportion was higher. The *r*_*j*_(*t* − Δ_*d*_, *t*) and *i*_*j*_(*t* − Δ_*d*_, *t*) scoring functions had a bad performance for such low levels of vaccinated herds, performed better for medium levels of vaccination, and performed worse when the proportion of vaccinated herds was higher than 0.2. In particular, *i*_*j*_(*t* − Δ_*d*_, *t*) was better than greedyV_infherds when the proportion of vaccinated herds was less than 0.2. On the contrary, as the proportion of vaccinated herds was higher, *r*_*j*_(*t*) performs better. Indeed, when less than 25% of herds were vaccinated, it was just as bad as the random scoring function, which was the second worse score after *s*_*j*_(*t*). But for a proportion of vaccinated herds higher than 0.45, its performance was close to the one of the best-performing greedy scores. Even when this proportion was more than 0.6, only *r*_*j*_(*t*), *i*_*j*_(*t*) and the greedy scores arrived to eradicate the disease.

Regarding the mean proportion of wasted doses (over time and over runs), it was almost zero when the vaccine allocation was done using *s*_*j*_(*t*) as criterion, irrespective of the proportion of vaccinated herds. The other scoring functions led to quite similar levels of wasted doses, except for *r*_*j*_(*t*) and *r*_*j*_(*t* − Δ_*d*_, *t*), which led to the highest proportion of wasted doses, particularly when the proportion of vaccinated herds was lower than 0.15.

In figure 4*b*, we observe that when the resource to allocate was a treatment, greedyT_infanimals managed to eradicate the disease when it was possible to treat at least 25 herds per decision time-step, i.e. 0.5% of the total number of herds. As for *i*_*j*_(*t*), it could eradicate the disease when this percentage was higher than 0.6%, and *i*_*j*_(*t* − Δ_*d*_, *t*) when it was at least 0.7% herds. When *b*_fix_ was equal to 0.9% of the total number of herds, greedyT_infherds_threshold, the random scoring function and *r*_*j*_(*t* − Δ_*d*_, *t*) also eradicated the disease, in that order. The other epidemiological scoring functions and the topological and demographic ones performed worse than the random score for all values of *b*_fix_. In particular, sales_*j*_(*t* − Δ_*d*_, *t*) only eradicated the disease if *b*_fix_ was higher than 1% of the total number of herds, *s*_*j*_(*t*) could only do it when this percentage was higher than 1.2%, and the other two scoring functions (*r*_*j*_(*t*) and pagerank_*j*_) eradicated it when it was possible to treat at least 1.3% and 1.4% of the total number of herds, respectively.

As for the number of used doses of treatment, they were in general lower for bad-performing scoring functions, and higher for those that performed the best. The exception was *r*_*j*_(*t*), which performed badly and led to a high number of used doses. When *b*_fix_ was high, it used even more doses on average than the best scoring functions for reducing disease prevalence.

Results in the epidemic scenario (electronic supplementary material, figure S6) were quite similar. Yet, for the treatment, only greedyT_infanimals, *i*_*j*_(*t*) and *i*_*j*_(*t* − Δ_*d*_, *t*) were always better than the random score. The greedyT_infherds_threshold indicator was better than the random score only when *b*_fix_ was less than 0.8% of the total number of herds. Additionally, electronic supplementary material, figure S7, shows that the variability of the percolation results were moderate between runs, with no or little overlapping 90% confidence intervals.

In addition, electronic supplementary material, figure S8, shows that when vaccine efficacy is decreased, the loss of performance is not the same for all scores. In particular, the scores that are not the best but perform rather well (epidemiological scores and greedyV_infherds) are the ones for which performance is most depreciated. This results in sales_*j*_(*t* − Δ_*d*_, *t*) performing better than *i*_*j*_(*t* − Δ_*d*_, *t*) when the average proportion of vaccinated herds was 55% and vaccine efficacy was 0.9 or less. Yet, despite the overall loss in efficiency, two of the greedy scores and one epidemiological score (*i*_*j*_(*t*)) still performed the best.

Finally, electronic supplementary material, figures S9 and S10, show that a disease with higher early peak and smaller infection duration (*β*/*γ* = 4, 1/*γ* = 30 days) spreads and fades out rapidly at the intra-herd level. At the same time, recurrent outbreaks are observed which are likely due to reintroduction of infected animals by trade. Electronic supplementary material, figure S11, shows that, in such a case, allocating vaccines according to the historic sales, in particular according to sales_*j*_(*t* − Δ_*d*_, *t*), was among the best strategies in both the epidemic and endemic scenarios.

## 4. Discussion

To control an infectious disease that spreads in a metapopulation network, allocating a limited resource is a fundamental yet difficult question, especially for large networks. In this study, we considered this resource allocation problem for a livestock disease that spreads over a large animal trade network, where the intra-herd infection and demographic dynamics was specified as an SIR stochastic model taking into account animal movements and demography.

The problem of resource allocation in networks had been previously addressed from several perspectives such as optimal control [23] and reinforcement learning [35], but mostly for networks where each node is an individual [10], or where the network is rather small [36]. Yet, in the context of a very large network these methods lack scalability for tracking the optimal solution [30]. In this work, we chose to concentrate on strategies based on scoring functions, heuristic and optimized, which consist of ordering the nodes of the network according to their score and allocating the resource to the top of the ranking, up to the limit given by the available resource.

First, following the greedy approach in [30], we provided new analytic scoring functions for controlling the disease spread over the animal metapopulation network by optimizing approximated objective functions. The scoring functions we derived depend on the infection-related state of the herd, and some are also dependent on the topology of the metapopulation network. They differ according to the objective of the control (minimizing the number of infected animals versus minimizing the number of infected herds) and the type of available resource (a protective vaccine or a treatment that reduces the infectious period). Meanwhile, most similar existing approaches for other population structures derive strategies solely for distributing a vaccine [5,11], or are concerned with only one objective to be optimized [10,13].

Through intensive simulations, we observed that these analytically obtained scoring functions can be optimal for reducing disease prevalence in the metapopulation, though this is not always the case. For example, even if greedyV_infanimals (the score for greedily minimizing a function on the total number of infected animals in the metapopulation) showed the greatest reduction in disease prevalence through vaccination, allocating vaccines according to the proportion of infected animals by herd, *i*_*j*_(*t*), can be just as good when the number of available vaccine doses equalled 25% of the initial number of animals in the metapopulation (figure 3). Even more, we observed that this was the case as long as the number of available doses was more than 15% of the initial number of animals in the metapopulation (figure 4*a*).

For the treatment, most of the scoring functions, in particular topological and demographic ones, were counterproductive in the sense that they performed worse than randomly allocating the resource among the infected herds (figures 3 and 4*b*). We explain this by the fact that the infected herds which were central in the network were not the most infected ones (in terms of the proportion of infected animals). Indeed, electronic supplementary material, figure S3*b*, shows that the random allocation among infected herds also targeted herds with many infected animals, while the scoring functions that performed badly only targeted high sized infected herds but generally with few infected animals.

Furthermore, we noticed that irrespective of the resource type, the optimized scoring function for an objective on the number of infected herds was outperformed by the optimized scoring function for an objective on the number of infected animals. Even if for vaccination, a slightly modified version of greedyV_infherds provided results almost as good as the ones of greedyV_infanimals (figures 3 and 4*a*), this was not the case for the treatment. Indeed, the allocation implemented using the greedyT_infanimals scoring function, i.e. the number of infected animals per herd, yielded undoubtedly the best results (figures 3 and 4*b*). This is probably due to the fact that the scoring functions for minimizing an objective on the number of infected herds only focus on the fast recovery of slightly infected herds (0 < *I*_*j*_(*t*) < 20), for vaccination, or on avoiding that completely healthy herds receive infectious animals (*I*_*j*_(*t*) > 0), for the treatment. Although this is the best way to have a small incremental number of infected herds from one instant to another according to these scoring functions, it does not take into account new animal infections, which only occur at the intra-herd level once the herd is infected. Our interpretation is that a scoring function obtained with the greedy approach (which consists of focusing on the short-term behaviour of the objective function) performs better for limiting the disease spread if the objective function it is built on directly captures the intra-herd aspect of the disease dynamics. Hence, minimizing a first-order approximation for an objective that directly concerns the number of infected animals can provide more performing scoring functions than that obtained by minimizing a higher-order approximation for an objective that does not.

Numerical investigations also allowed evidencing that intra-herd health information can be crucial for optimally controlling the propagation of a slowly spreading disease such as the one considered in this work. In most combinations, given by the resource’s type and available quantity, even if topology-based scoring functions managed to limit disease spread, scoring functions based on the infection-related state of herds performed better (figure 4; electronic supplementary material, figure S6). This observation can give some insight into why most control strategies implemented in real systems might fail to eradicate livestock diseases in areas that lack this kind of information.

A final interesting remark is that the best scoring functions for reducing disease prevalence can induce a higher number of wasted vaccine doses or a higher number of used treatment doses compared to other scores that performed less well for controlling the disease spread (figure 4; electronic supplementary material, figures S3 and S6). However, for vaccination, they are not necessarily those that vaccinate the highest proportion of herds.

To our knowledge, our work is one of the few studies that explores dynamic resource allocation in a metapopulation network for many allocation scoring functions (16 heuristics and at least 2 optimized scores by measure), while varying the available quantity of the resource in two different scenarios. Despite the fact that the performances of the scores could have been different if the network had been static or not scale-free, score-based resource allocation can be a relevant approach for controlling pathogen spread in other cases, as the complexity of the problem is mainly due to the large dimension of the network. Furthermore, we stress that the scores we found by optimization using the greedy approach are the same irrespective of the network topology, as they are based on fixed trade parameters representing the static aggregated network. Indeed, they are built on a static view of the network, irrespective of whether it is actually static or dynamic (as it is in our study). We believe it would be possible to use the same approach to obtain optimized scores that take into account the dynamic nature of the network, i.e. scores that are function of time-dependent trade parameters, although this requires a new formal analysis. Of course, these new scores could be of a different form from the ones we found, and hence their performance might also be different.

We assessed the robustness of the results regarding vaccine allocation by considering realistic values for the vaccine efficacy. This showed a limited impact on the relative performance of the different scoring functions (electronic supplementary material, figure S8). We note that although in reality vaccines are rarely perfectly effective, and also take some time to be effective, it did not seem straightforward to determine an appropriate time frame for the vaccine to have an effect. More importantly, it seems unlikely that a slight delay in the effect of the vaccine would have a significant impact on our results, given that we considered a pathogen that spreads rather slowly.

Regarding the limitations of our work, we emphasize that in the context of a fast-spreading pathogen, the current framework is not really appropriate. In such a case, other decision factors should be taken into account. For example, if the disease is zoonotic or has a strong economic impact, the social planner may consider more radical options, such as mass culling. In this case, the question of resource allocation thus becomes irrelevant. In particular, we showed that for a disease with higher early peak and shorter infection duration (*β*/*γ* = 4, 1/*γ* = 30 days) an intra-herd epidemic extinguishes before a new disease introduction occurs (electronic supplementary material, figures S9 and S10). Therefore, it is not surprising that vaccinating herds that sell many animals appears as a good strategy for limiting disease propagation (electronic supplementary material, figure S11). So, the resource allocation problem seems more straightforward in such a scenario and does not necessarily require an optimization procedure.

Additionally, a parameter that could impact our conclusions is the decision step, Δ_*d*_, for which we considered a fixed heuristic value. Although the assumption of regular vaccination decisions defined by the duration of the protection conferred by the vaccine appears to be a realistic hypothesis relative to field practice, a more versatile assumption could be considered to determine the frequency of allocation decisions. Indeed, the decision step could be determined in an adaptive manner by the social planner, for example by taking into account the stability in disease prevalence, or some external input such as the farmers’ demand for accelerating resource allocation. A second option would be to determine the decision step by optimization. Yet, these are essentially different problems from the one we addressed in this article: determining when to allocate instead of where to allocate a limited resource. Optimizing both aspects at the same time is a more complex problem that, to the best of our knowledge, has only been addressed by heuristic approaches [37]. In particular, it does not seem straightforward to address with the approach of this study.

Finally, we stress that the performance of the epidemiological and the greedy scoring functions can be counterbalanced by their difficulty of access. Indeed, having updated knowledge on the epidemiological state of all the herds of the network is a strong hypothesis in real life, as this kind of information can be hard to gather for most livestock diseases [38]. For example, a scoring function calculated as the increment in the proportion of infected animals in a herd over a certain period can be observed through changes in the herd’s seroprevalence between two time points, which incurs into increased logistics, can be observed with error and not in real time. Furthermore, having such updated and detailed health-related information can be costly, and this cost should be taken into account in the constrained optimization problem for the allocation. Among the possible perspectives of this work, the previous point opens an important one: combining scoring functions for improved performances, and above all for yielding a scoring function that can be useful in practice. This could be achieved, for example, through the (linear) combination of scoring functions, or through the selection of herds at the top of the ranking given by several scoring functions that do not allocate the resource in a similar way. Additionally, for cases when the value of the score is the same for many herds, the allocation could be done using a second scoring function that would take different allocation decisions. As a second, more methodological perspective, the greedy scoring functions built on first-order approximations could be eventually constructed using higher-order approximations. This could lead to analytic scores that also depend on the network topology, and could improve their performance. Although it has to be stressed that they were already among the best-performing scores for reducing disease prevalence in all cases. Finally, despite the fact that we focused on a protective vaccine and a treatment that increases the recovery rate, other types of resources could be studied with the same approach. However, it might not be straightforward to derive the analytic expression of the greedy scoring function in such cases. For example, the effect of the restriction of animal movements, which is a relevant control measure in this context, lies on the connections of the herd rather than on the intra-herd level, which could further complicate the derivation of the scoring function.

## Data Availability

Additional figures, details on the greedy approach and on data generation are provided in electronic supplementary material [39]. The Python simulation code for generating data and figures is available at https://github.com/CristanchoLina/DRAAnimalMetapop.
